# Manipulation of the endocytic pathway and phagocyte functions by *Mycobacterium tuberculosis* lipoarabinomannan

**DOI:** 10.3389/fcimb.2014.00187

**Published:** 2015-01-12

**Authors:** Isabelle Vergne, Martine Gilleron, Jérôme Nigou

**Affiliations:** ^1^Institut de Pharmacologie et de Biologie Structurale, Centre National de la Recherche ScientifiqueToulouse, France; ^2^Institut de Pharmacologie et de Biologie Structurale, Université Toulouse III - Paul SabatierToulouse, France

**Keywords:** lipoarabinomannan, *Mycobacterium*, cytokine, phagosome, apoptosis, autophagy

## Abstract

Lipoarabinomannan is a major immunomodulatory lipoglycan found in the cell envelope of *Mycobacterium tuberculosis* and related human pathogens. It reproduces several salient properties of *M. tuberculosis* in phagocytic cells, including inhibition of pro-inflammatory cytokine production, inhibition of phagolysosome biogenesis, and inhibition of apoptosis as well as autophagy. In this review, we present our current knowledge on lipoarabinomannan structure and ability to manipulate the endocytic pathway as well as phagocyte functions. A special focus is put on the molecular mechanisms employed and the signaling pathways hijacked. Available information is discussed in the context of *M. tuberculosis* pathogenesis.

## Introduction

*Mycobacterium tuberculosis* (*M.tb*), the causative agent of tuberculosis, is one the most effective human pathogens. Its virulence is multifactorial but initially relies on its ability to parasite and manipulate phagocytic cells in the lung. Mannose-capped lipoarabinomannan (ManLAM), a macroamphiphilic lipoglycan exposed at the surface of *M.tb* cell envelope (Nigou et al., 2003; Pitarque et al., 2008), is a key factor allowing the bacilli to manipulate phagocyte functions (Chatterjee and Khoo, 1998; Gilleron et al., 2008). Indeed, it reproduces several salient properties of *M.tb* in phagocytic cells, including inhibition of pro-inflammatory cytokines production, inhibition of phagosome maturation, inhibition of macrophage apoptosis, and inhibition of autophagy. ManLAM is a Pathogen-Associated Molecular Pattern recognized by several receptors of the innate immune system, including the C-type lectins Mannose Receptor (MR), DC-SIGN and Dectin-2, as well as TLR2 (Gilleron et al., 2008; Ray et al., 2013). It is a potential ligand for the entry of *M.tb* into macrophages *via* the MR (Schlesinger et al., 1994) and into dendritic cells (DCs) *via* DC-SIGN (Maeda et al., 2003; Tailleux et al., 2003). ManLAM inhibitory properties mainly rely on its ability to bind these two lectins. ManLAM can be a ligand of these receptors not only at the surface of *M.tb* bacilli but also as a soluble molecule. Indeed, it is delivered from infected macrophages, through exosomes or apoptotic vesicles, to non-infected bystander phagocytic cells (Beatty et al., 2000; Schaible et al., 2003). This pathway is thought to be critical for shaping immune response but might also be used by the pathogen as a way to disseminate immunomodulatory molecules such as ManLAM.

## Lipoarabinomannan structure and physiological role

**Figure 1:**
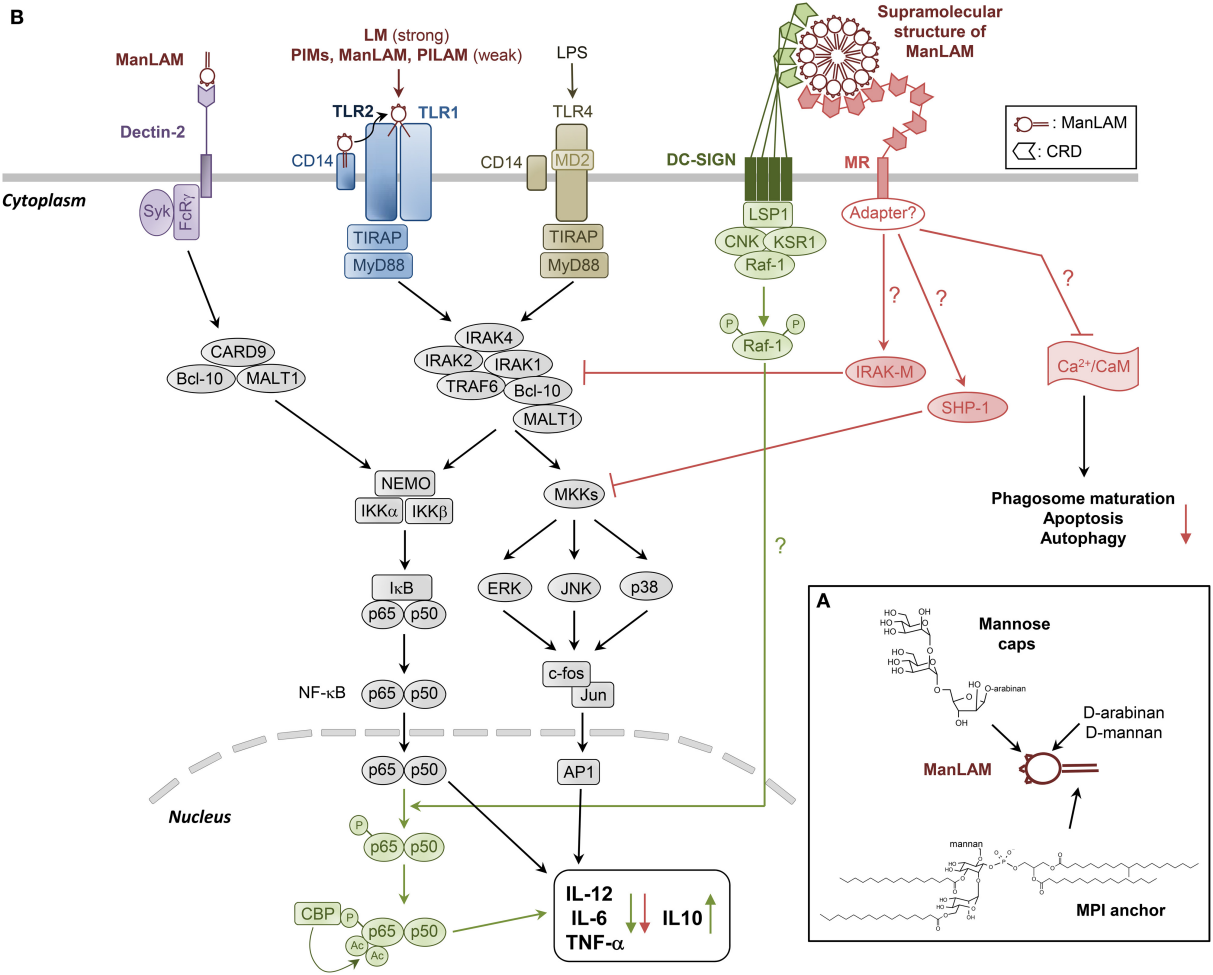
**Structural model of and cell signaling pathways triggered by ManLAM and its biosynthetic precursors LM and PIMs**. **(A)** ManLAM is a 17 kDa heterogenous macromolecule exhibiting a tripartite structure: (i) a MPI anchor, which can be mono- to tetra-acylated, (ii) a polysaccharide backbone composed of D-Mannan and D-Arabinan, and (iii) mannose caps, which are mono-, (α1→2)-di- and (α1→2)-tri-mannoside units. PIMs and LM are biosynthetic precursors of LAM. Their structure is based on the MPI anchor, glycosylated by one to six mannose units (PIMs) or the full mannan domain (LM). To our present knowledge, the MPI anchor and the Mannose caps are the main structural determinants of ManLAM biological properties. **(B)** LM, and to a much lesser extent PIMs, PILAM and ManLAM, induce pro-inflammatory cytokines production in DCs and macrophages *via* the recognition of tri- or tetra-acylated MPI anchor by TLR2/TLR1 heterodimer. ManLAM elicits cytokines in bone marrow-derived DCs *via* mannose caps binding to Dectin-2. But it also inhibits the production of pro-inflammatory cytokines IL-12, TNF-α, and IL-6, and induces IL-10 by LPS-stimulated human DCs through DC-SIGN ligation. The signaling pathway involves activation of Raf-1, which results in the phosphorylation of the p65 subunit of NF-κB at Ser276, leading to the acetylation of p65 by two histone acetyltransferases. Translocation of NF-κB in response to TLR activation, and initially dedicated to the transcription of the pro-inflammatory cytokine-coding genes, is reoriented on anti-inflammatory promoter targets, resulting in the decrease of these cytokines to the benefit of IL-10. ManLAM also inhibits IL-12 and TNF-α in macrophages independently of IL-10 production, by directly acting on the TLR4 signaling cascade through induced expression of IRAK-M, which can compete with IRAK1 for binding to TRAF6 and thus inhibit NF-κB activation. ManLAM also promotes tyrosine dephosphorylation of multiple proteins including MAPK, an effect that might be explained by an increased activity of tyrosine phosphatase SHP-1. MR is likely to mediate ManLAM immunosuppressive activities in macrophages, although it has no signaling motif in its cytoplasmic domain, raising the intriguing question as to whether it associates with adapter molecules to transduce signals. The ability of ManLAM to bind MR might in part determine its other inhibitory properties as detailed in Figure 2.

Lipoarabinomannan (LAM) is ubiquitously found in mycobacterial species (Nigou et al., 2003; Briken et al., 2004; Gilleron et al., 2008; Mishra et al., 2011; Angala et al., 2014). It presents a tripartite structure including a lipid anchor, namely Mannosyl-Phosphatidyl-*myo*-Inositol (MPI), a polysaccharide backbone composed of D-Mannan and D-Arabinan, and finally caps (Figure 1A). MPI anchor is based on a *sn*-glycerol-3-phospho-(1-D-*myo*-inositol) unit with one α-D-Mannopyranosyl (α-D-Man*p*) unit linked at *O*-2 of the *myo*-inositol. Four potential sites of acylation are present on the anchor: positions 1 and 2 of the glycerol unit, position 6 of the Man*p* unit and position 3 of the *myo*-inositol (Nigou et al., 1999; Gilleron et al., 2000) (Figure 1A). LAM and its biosynthetic precursors, phosphatidyl-*myo*-inositol-mannosides (PIMs) and lipomannan (LM), are predominantly tri- and tetra-acylated by palmitic and tuberculostearic (10-methyl-octadecanoic) acids (Khoo et al., 1995; Gilleron et al., 1999). Position *O*-6 of *myo*-inositol is glycosylated by the mannan core. PIMs comprise different glyco-forms, containing one to six α-D-Man*p* units (PIM_1_ to PIM_6_), PIM_2_ and PIM_6_ being the most abundant ones. The D-mannan core of LAM and LM is composed of an (α1→6)-Man*p* backbone substituted at some *O*-2 by a single α-D-Man*p* unit. The D-arabinan portion of LAM contains about 60 arabinofuranosyl (Ara*f*) units which are present as a single arabinan chain attached through an (α1→2) linkage near the middle of the D-mannan core (Kaur et al., 2014). The innermost region is made of a linear (α1→5)-Ara*f* backbone and is followed by a branched region. The non-reducing termini consist of branched hexa-arabinofuranosides and linear tetra-arabinofuranosides, which end with an Ara*f*-(β1→2)-Ara*f*-(α1→ motif. Some β-Ara*f* units are substituted at *O*-5 by capping motifs. The caps differ according to the mycobacterial species. LAM from slow-growing mycobacteria, including the pathogenic species *M.tb*, *Mycobacterium leprae* and *Mycobacterium ulcerans*, are capped with mono-, (α1→2)-di- and (α1→2)-tri-mannoside units (mannose-capped LAM is referred to as ManLAM) (Chatterjee et al., 1992) (Figure 1A). In contrast, LAM from fast-growing species is either capped by phospho-*myo*-inositol units (PILAM), such as in the non-pathogenic model organism *Mycobacterium smegmatis*, or not capped (AraLAM). LAM in any strain further displays considerable structural micro-heterogeneity, with various acyl-forms and glyco-forms. In addition, ManLAM may be substituted by discrete motifs, such as succinyl residues on the arabinan chain or (α1→4)-linked methyl-thio-D-xylose (MTX) residues on some terminal Man*p* units of the mannose caps or the mannan core.

LAM is not restricted to mycobacteria. Indeed, LAM-like molecules are also produced by phylogenetically close relatives of bacteria of the suborders *Corynebacterineae* and *Pseudonocardineae*, including *Corynebacterium* (Tatituri et al., 2007), *Rhodococcus* (Garton et al., 2002; Gibson et al., 2003b), *Tsukamurella* (Gibson et al., 2004), *Turicella* (Gilleron et al., 2005), *Amycolatopsis* (Gibson et al., 2003a), or *Saccharothrix* (Gibson et al., 2005) genera. In these bacteria, lipoglycans are thought to functionally replace lipoteichoic acid otherwise produced by low G+C Gram-positive bacteria. These macroamphiphiles play a fundamental role in the physiology of bacteria, although yet not fully understood (Ray et al., 2013). Defective or deficient lipoglycans synthesis is associated with lethality or growth defects (Gilleron et al., 2008) and changes in lipoglycan structures have a significant impact on the cell wall integrity of mycobacteria (Fukuda et al., 2013). For example, structural defects in LM and LAM in *M. smegmatis* result in loss of acid-fast staining, increased sensitivity to β-lactam antibiotics, and faster killing by macrophages (Fukuda et al., 2013). Accordingly, mycobacterial D-arabinan biosynthesis is the target of ethambutol (Deng et al., 1995), a first-line drug in the treatment of tuberculosis, as well as of benzothiazinones (Makarov et al., 2009), which are new antituberculous drug candidates in preclinical development. The elucidation of the complete biosynthetic pathways of these important molecules is therefore expected to afford novel therapeutic targets (Angala et al., 2014).

In the context of host-pathogen interaction, to our present knowledge, the MPI anchor and the mannose caps are the main structural determinants of ManLAM biological properties, the role of the discrete motifs remaining elusive. The MPI anchor is recognized by TLR2/TLR1 heterodimer, whereas the mannose caps allow the binding to C-type lectins (Gilleron et al., 2008; Ray et al., 2013).

## Manipulation of phagocytes responses

The success of *M.tb* as an intracellular pathogen relies on its extraordinary capacity to disarm phagocyte antibacterial defenses whereby turning hostile phagocytes into safe havens for replication. Beyond their impact on innate immune responses such manipulations can also be detrimental in development of an efficient adaptive immunity. Interestingly, several of these manipulations can be mirrored by purified ManLAM.

### Pro-inflammatory cytokines production

The inflammatory response is crucial to control *M.tb* infection through macrophage activation and granuloma formation. LM, and to a much lesser extent PIMs, PILAM, and ManLAM, induce pro-inflammatory cytokines production *via* the recognition of tri- or tetra-acylated MPI anchor by TLR2/TLR1 (Gilleron et al., 2003, 2006; Vignal et al., 2003; Quesniaux et al., 2004; Nigou et al., 2008; Ray et al., 2013) (Figure 1B). However, a prolonged stimulation of TLR2 has been shown to result in inhibition of MHC class II transactivator expression, MHC class II molecule expression and antigen presentation (Gehring et al., 2004). *M.tb* might have subverted this general mechanism of negative-feedback regulation that prevents excessive T cell-mediated inflammation to evade recognition by CD4^+^ T cells (Harding and Boom, 2010).

ManLAM was recently shown to elicit TNF-α, IL-6, and IL-10 in bone marrow-derived DCs *via* mannose caps binding to Dectin-2 (Yonekawa et al., 2014) (Figure 1B). However, we previously found that *M.tb* ManLAM can also inhibit the production of pro-inflammatory cytokines IL-12 and TNF-α by LPS-stimulated human DCs (Nigou et al., 2001, 2002) (Figure 1B). We initially proposed the C-type lectin MR to mediate ManLAM inhibitory activity because the latter (i) relied on the presence of both the mannose caps and the fatty acids which are also required for ManLAM binding to MR and (ii) could be mimicked by an agonist anti-MR monoclonal antibody. However, it was later shown that ManLAM binding to DCs is not inhibited by anti-MR but rather by anti-DC-SIGN antibodies and that a blocking anti-DC-SIGN antibody inhibits ManLAM-induced IL-10 production by LPS-stimulated DCs (Geijtenbeek et al., 2003). Why ManLAM only binds DC-SIGN on DCs, although MR is expressed on these cells, remains unclear (Blattes et al., 2013). ManLAM also inhibits IL-12 and TNF-α in human THP-1 (Knutson et al., 1998) and murine RAW 264.7 (Pathak et al., 2005) macrophage cell lines although DC-SIGN is absent. MR is likely to mediate ManLAM effect in these cells, as the ability of MR to trigger an anti-inflammatory signal was confirmed by other independent studies (Chieppa et al., 2003; Zhang et al., 2005).

How DC-SIGN or MR signal into the cells and interfere with LPS-induced TLR4 signaling is not yet completely understood (Figure 1B). DC-SIGN displays intracellular motifs that are able to constitutively recruit the lymphocyte-specific adaptor protein LSP1 which associates the complex KSR1-CNK-Raf-1 (Gringhuis et al., 2009) (Figure 1B). Upon ligand binding, activation of Raf-1 results in the phosphorylation of the p65 subunit of NF-κB at Ser276, leading to the acetylation of p65 (Gringhuis et al., 2007). NF-κB activity is then prolonged and increases the transcription rate at the IL-10 anti-inflammatory cytokine promoter. However, Raf-1 signaling alone does not induce cytokine expression. Translocation of NF-κB in response to TLR activation and initially dedicated to the transcription of the pro-inflammatory IL-12p35, IL-12p40, IL-6, and TNF-α cytokine-coding genes is reoriented on anti-inflammatory promoter targets, resulting in the decrease of these cytokines to the benefit of IL-10 (Gringhuis et al., 2007) (Figure 1B). Gringhuis et al. (2009) proposed that DC-SIGN may discriminate among mannosylated and fucosylated ligands and modulate the TLR signaling into a pro- or anti-inflammatory response respectively. However, this appears to be in contradiction with the set of data showing that ManLAM or synthetic mannosylated analogs engaging DC-SIGN inhibit pro-inflammatory cytokines production (Nigou et al., 2001; Geijtenbeek et al., 2003; Blattes et al., 2013). MR has no signaling motif in its cytoplasmic domain, raising the intriguing question as to whether it associates with adapter molecules to transduce signals. Pathak et al. (2005) demonstrated that ManLAM dampens IL-12 in RAW 264.7 macrophages independently of IL-10 production, by directly acting on the TLR4 signaling cascade through induced expression of IRAK-M, which can compete with IRAK1 for binding to TRAF6 and thus inhibit NF-κB activation (Figure 1B).

ManLAM anti-inflammatory activity relies on its ability to bind DC-SIGN or MR and both the mannose caps and the fatty acids are required for efficient binding (Nigou et al., 2001, 2002). Indeed, fatty acids induce a supramolecular organization of ManLAM in aqueous solution, resulting in the formation of a 30 nm spherical structure (Figure 1B), composed of approximately 450 molecules with the mannose caps exposed at the surface (Riviere et al., 2004). This multivalent supramolecular structure allows multipoint attachment of ManLAM, *via* mannose caps, to the Carbohydrate Recognition Domains (CRD) of multimeric DC-SIGN receptors (Feinberg et al., 2001; Mitchell et al., 2001), thereby ensuring high affinity binding (Nigou et al., 2001; Riviere et al., 2004) (Figure 1B). Following this rationale, we were able to design fully synthetic compounds mimicking the bioactive supramolecular structure of ManLAM, i.e., mannodendrimers, that display potent anti-inflammatory activity both *in vitro* and *in vivo* and that could be of therapeutic use (Blattes et al., 2013).

The ability of ManLAM to bind MR might in part determine its other inhibitory properties as described below (Figure 1B) (Gilleron et al., 2008).

### Phagosome maturation

One main function of professional phagocytes is the uptake of microorganisms through phagocytosis. This event results in formation of a vacuole called phagosome which then matures into a phagolysosome through a series of fusion reactions with the endocytic and secretory pathways and ultimately fusion with lysosomes (Flannagan et al., 2012). Maturation endows phagosome with new bactericidal properties predominantly hydrolase activities, acidic pH and antimicrobial peptides. Therefore, phagosome maturation process is crucial for killing of captured microbes as well as their antigen presentation to T lymphocytes. Inhibition of phagosome maturation by *M.tb* was reported more than 40 years ago (Armstrong and Hart, 1971). Since then, numerous mycobacterial factors have been identified and characterized as disruptors of phagosome maturation (Russell, 2011), including ManLAM and PIMs (Fratti et al., 2001; Vergne et al., 2004). Importantly, the ability of *M.tb* to block phagosome maturation is shared by other pathogenic mycobacteria such as *Mycobacterium avium* and *Mycobacterium marinum* which produce ManLAM but not by non-pathogenic *M. smegmatis* which produces PILAM (Anes et al., 2006; Appelmelk et al., 2008; de Chastellier et al., 2009).

Early work by Deretic and colleagues showed that mycobacteria block phagosome maturation between stages orchestrated by Rab5 and Rab7, two small GTPases involved in membrane trafficking and present on early and late endosomes, respectively (Via et al., 1997). Later on, they pinpointed this block to impairment in recruitment of EEA1, a tethering protein and Rab5 effector, essential for phagosome maturation (Fratti et al., 2001). EEA1 recruitment is instrumental in delivering hydrolases such as Cathepsin D and H^+^-ATPase subunit Vo from Trans-Golgi-Network (TGN) to the phagosome (Fratti et al., 2003). EEA1 is recruited to the phagosomal membrane via Rab5 and phosphatidylinositol 3-phosphatase (PI3P) which is synthesized by type III PI3Kinase, hVPS34 (Fratti et al., 2001). In the same report, authors showed that ManLAM-coated beads, in contrast to control beads, prevent EEA1 recruitment to the phagosomal membrane, delivery of Cathepsin D, and phagosome acidification (Fratti et al., 2001, 2003). Inhibition of phagosome maturation by ManLAM was later confirmed by several groups (Hmama et al., 2004; Kang et al., 2005; Welin et al., 2008).

**Figure 2:**
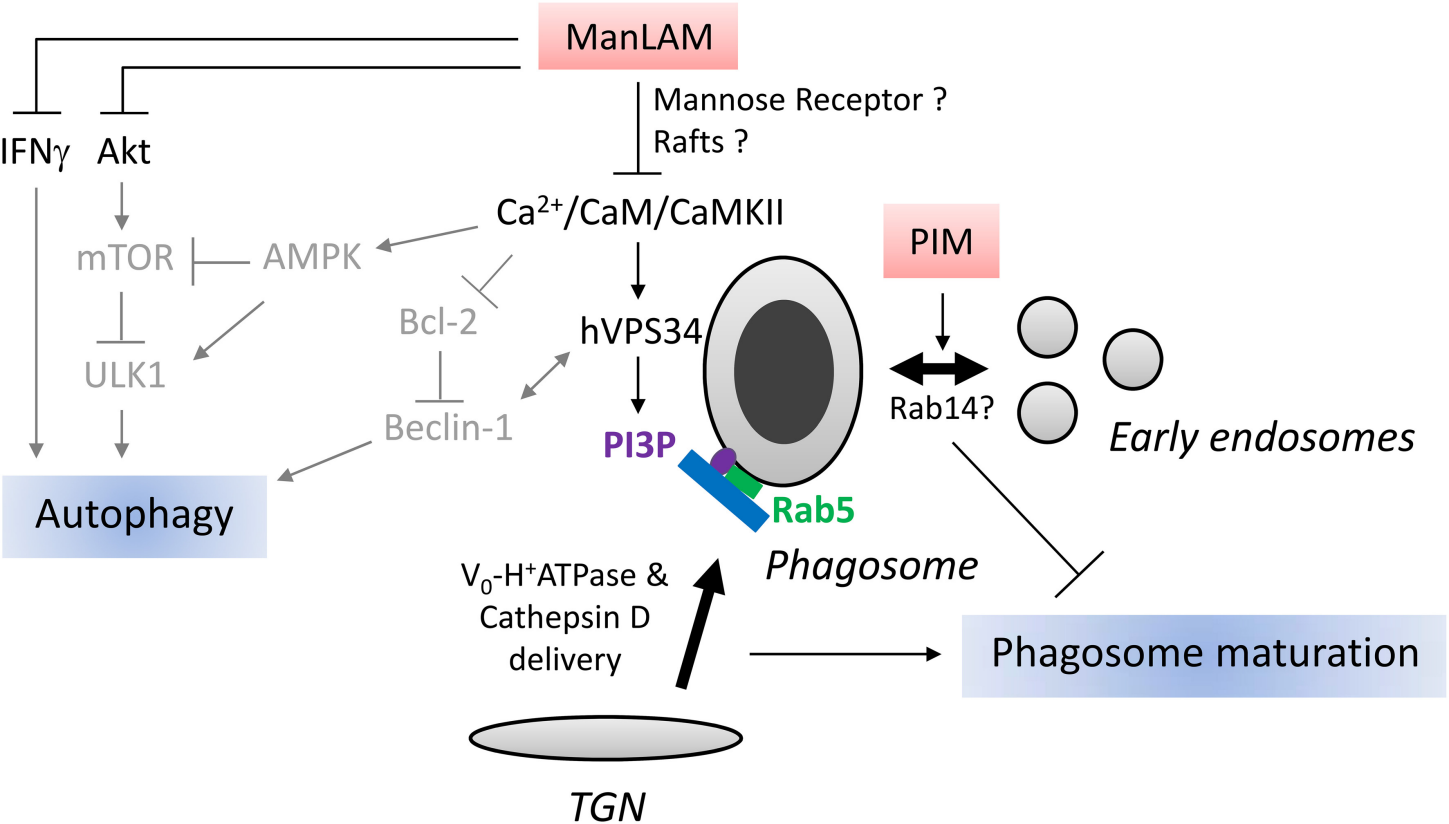
**Schematic representation of ManLAM and PIM action on phagosome maturation and autophagy. Right part:** After phagocytosis, mycobacteria reside in a vacuole, called phagosome. Phagosome maturation consists in a series of fusion events with exocytic and endocytic pathways. One key step is the delivery of Cathepsin D and H^+^-ATPase subunit Vo from Trans-Golgi-Network (TGN) to the phagosome. This step is mediated by tethering protein EEA1 which is recruited to the phagosome by small GTPase Rab5 and phosphatidylinositol 3-phosphate (PI3P). ManLAM blocks phagosome maturation through inhibition of Ca^2+^/CaM/CaMKII signaling pathway involved in PI3P production by type III PI3Kinase hVPS34. ManLAM can also block phagosome maturation by engaging mannose receptor and disrupting membrane microdomains, rafts, however, the link with Ca^2+^/CaM signaling has not been studied. PIMs, ManLAM precursor, impair phagosome maturation by stimulating fusion between phagosome and early endosomes. *Mycobacterium tuberculosis* recruits Rab14 to phagosome to promote early endosome fusion thus impairs phagosome maturation. It remains to be established whether PIMs promotes early endosome fusion through Rab14 recruitment. **Left part:** Mammalian target of rapamycin (mTOR) kinase, activated by Ser/Thr kinase Akt and inhibited by AMP-activated Protein Kinase (AMPK), is a master repressor of autophagy. Beclin-1, an autophagy-related protein in complex with hVPS34, is essential for autophagy. Beclin-1/hVPS34 complex is activated by AMPK and repressed by Bcl-2. ULK1, another important autophagy-related protein, is activated by AMPK and inhibited by mTOR. Ca^2+^ influx has been shown to activate AMPK, hVPS34, and represses Bcl-2 expression. IFNγ induces autophagy. Based on known effects of ManLAM on Ca^2+^ influx, Bcl-2, Akt and IFNγ signaling, we postulate that ManLAM might inhibit autophagy by targeting Beclin-1/hVPS34 complex, Akt/mTOR or IFNγ pathways. The relationship between effects of ManLAM on these different signaling pathways and autophagy awaits investigation. Arrows and characters are represented in gray to indicate that the molecular mechanisms of LAM action on autophagy are hypothetical.

Another important player in phagosome maturation is Ca^2+^ signaling. Phagocytosis of dead *M.tb* but not of live *M.tb* triggers an increase of cytosolic Ca^2+^ that results in activation of calmodulin-dependent kinase II (CaMKII) (Malik et al., 2000, 2001). Inhibition of Ca^2+^, Calmodulin (CaM) and CaMKII prevents phagosome containing dead *M.tb* to fuse with lysosomes. Vergne et al. showed that Ca^2+^ signaling is central for PI3P synthesis on phagosomal membrane, consequently for EEA1 recruitment (Vergne et al., 2003). CaM and CaMKII seem to play a role in hVPS34 recruitment and/or activation. Notably, in contrast to PILAM, ManLAM limits Ca^2+^ influx in cytosol, thus explaining its effect on EEA1 recruitment and phagosome maturation (Figure 2). Interestingly, PIMs can also inhibit phagosome acidification, not by preventing EEA1 recruitment, but by promoting fusion between phagosome and early endosomes (Vergne et al., 2004). How PIMs trigger early endosome fusion remains to be elucidated but it might involve Rab14, a small GTPase specifically recruited by live mycobacteria to favor phagosome-early endosomes fusion and block phagosome acidification (Kyei et al., 2006) (Figure 2).

What are ManLAM molecular targets, upstream of Ca^2+^ signaling, responsible for phagosome maturation arrest? Two main mechanisms, non-mutually exclusive, have been uncovered. Schlesinger's group has demonstrated that ManLAM limits phagosome maturation by binding to MR (Kang et al., 2005). Interestingly, ManLAM acyl chains are important to maintain this blockade beyond 1 h, suggesting a possible additional mechanism for ManLAM action. ManLAM can insert into lipid microdomains, called rafts, *via* the MPI anchor, resulting in membrane disorganization and inhibition of membrane fusion (Hayakawa et al., 2007; Welin et al., 2008). However, it is still unclear whether rafts disruption and/or MR are responsible of Ca^2+^ signaling inhibition or are completely independent mechanisms.

### Apoptosis

The role(s) of apoptosis and other cell-death pathways in Tuberculosis remain(s) a matter of intense debate. Several reports suggest that inhibition of excessive apoptosis may be beneficial for the pathogen during early stage of infection for maintaining its replicative niche and limiting cross-presentation and cross-priming of CD8^+^ T-cells through phagocytosis of apoptotic bodies by DCs. *M.tb* seems to be able to block both extrinsic pathway of host cell apoptosis which relies on activation of death receptors (Fas/CD95, TNFR1) and the intrinsic pathway triggered by mitochondrial outer membrane permeabilization (Briken and Miller, 2008). Although *M.tb* genes involved in these inhibitions are just beginning to be unveiled, ManLAM was one of the first mycobacterial product identified as an inhibitor of apoptosis (Rojas et al., 2000; Briken and Miller, 2008).

The mechanisms of apoptosis inhibition by ManLAM seem to be multiple. As for phagosome maturation, ManLAM inhibition of Ca^2+^ signaling appears to be an important step in blocking infection-induced apoptosis (Rojas et al., 2000). Numerous Ca^2+^-associated events are known to play a role in apoptosis, among them alteration of mitochondrial permeability transition and down-regulation of anti-apoptotic protein Bcl2 have been shown to be repressed by ManLAM (Rojas et al., 2000). Besides Ca^2+^ signaling, ManLAM can prevent intrinsic apoptosis pathway through activation of Ser/Thr kinase Akt and phosphorylation of the pro-apoptotic protein Bad (Maiti et al., 2001). More recently, one report indicates that ManLAM can promote extracellular release of soluble TNF-α receptor. Thus, ManLAM might also interfere with the extrinsic apoptosis pathway by neutralizing TNF-α (Richmond et al., 2012).

### Autophagy

Autophagy is a highly conserved eukaryotic intracellular process that carries out lysosomal degradation of damaged, superfluous or toxic cytoplasmic components (Levine et al., 2011; Rubinsztein et al., 2012). In addition to its housekeeping role, autophagy plays major immunological functions, especially, in host anti-bacterial defenses (Levine et al., 2011; Deretic et al., 2013). These functions range from effector of pattern recognition receptors and inflammation regulation to antigen presentation and direct elimination of microbial agents. Specifically, autophagy is a key immune effector involved in intracellular clearance of important bacterial pathogens such as *M.tb*.

Autophagy is orchestrated by more than 30 dedicated proteins, called autophagy-related proteins (Atg) (Marino et al., 2014). The autophagic process begins with formation of an isolation membrane initiated by Ser/Thr kinase Ulk1 (Atg1), which phosphorylates Beclin-1 (Atg6) in complex with hVPS34 to promote its activation (Russell et al., 2013). The isolation membrane is then expanded through action of two ubiquitin-like conjugation systems, the covalent linkage of Atg12 with Atg5 and of LC3 (Atg8) with phosphatidylethanolamine, which lead to engulfment of intracellular components inside a double-membrane bound organelle called autophagosome. LC3, along with entrapped cytosolic content, is then degraded after fusion of autophagosome with lysosomes. In the context of phagocytosis, a non-canonical autophagy pathway, called LC3-associated phagocytosis (LAP), has been described which involves direct LC3 lipidation on the phagosomal membrane (Mehta et al., 2014). This alternative pathway, triggered by some Pattern Recognition Receptors, such as TLR2, appears to be ULK1 independent and important in modulating innate immune response (Mehta et al., 2014). However, the detailed molecular mechanisms and the functional role(s) of LAP still remain to be fully elucidated.

*M.tb*, like other intracellular intracellular pathogens, has developed mechanisms to manipulate autophagic pathway (Huang and Brumell, 2014). Interestingly, Shui et al. showed that phagosomes containing ManLAM-coated beads display less LC3 than those containing PILAM-coated beads (Shui et al., 2011). Likewise, macrophage treatment with ManLAM for 24 h results in diminution of autophagy as seen by LC3 immunoblotting (personal observation). Autophagy-related proteins play major roles in mediating IFNγ-induced host defenses (Levine et al., 2011; Deretic et al., 2013). Since ManLAM can repress IFNγ responses, it is tempting to speculate that ManLAM might also interfere with autophagy in this context (Sibley et al., 1988; Chan et al., 1991). The action mechanism of ManLAM on autophagy has not been revealed yet, but based on its effect on hVPS34 in phagosome maturation one can postulate that it might inhibit autophagy by modulating hVPS34 in complex with Beclin-1 (Figure 2). In addition, Bcl-2 interacts with Beclin-1 to block autophagy, thus ManLAM might impair autophagy via upregulation of Bcl-2 expression (Rojas et al., 2000; Pattingre et al., 2005). ManLAM inhibition of Ca^2+^ influx could also affect the Ca^2+^/AMP-activated protein kinase (AMPK) signaling pathway involved in mammalian target of rapamycin (mTOR) kinase- and ULK1-dependent autophagy (Vergne et al., 2003; Alers et al., 2012). Alternatively, ManLAM might repress autophagy through activation of type I PI3Kinase and Akt (Maiti et al., 2001; Ravikumar et al., 2010). Further investigations are definitely required to better understand how ManLAM interferes with autophagy, whether it represses canonical autophagy and/or LAP and what is the significance of this inhibition in terms of phagosome trafficking and phagocyte survival.

## Conclusion

ManLAM, as a purified molecule, reproduces several salient inhibitory properties of *M.tb* in phagocytic cells. However, the role played by ManLAM in the context on an infection by *M.tb* remains unclear. ManLAM immunosuppressive activities rely on the presence of the mannose caps. But, an *M.tb* mutant lacking the mannose caps on LAM was not affected for its virulence in mice nor for its interaction with phagocytic cells *in vitro* (Appelmelk et al., 2008; Afonso-Barroso et al., 2012). In contrast, an aptamer against ManLAM was found to inhibit *M.tb* infection in mice and Rhesus monkeys (Pan et al., 2014). Moreover, protein LprG, which binds ManLAM and determines its cell surface localization, was found to be essential for virulence of *M.tb* and to control phagolysosomal fusion (Gaur et al., 2014; Shukla et al., 2014). These data are not necessarily contradictory. Indeed, the envelope of mycobacteria is exceptionally rich in mannoconjugates bearing (α1→2)-oligomannosides, including LM, PIM_6_, arabinomannan or mannoproteins that are able to bind C-type lectins (Pitarque et al., 2005; Torrelles and Schlesinger, 2010). Of note, LprG has been shown to bind LM and PIMs, in addition to ManLAM (Drage et al., 2010), suggesting that the role of LprG might not be attributable to ManLAM inhibitory activities only. Altogether, data converge to indicate that DC-SIGN/MR ligands are most probably redundant at the *M.tb* cell surface, possibly because targeting these receptors is mandatory for the pathogen to manipulate and survive inside the infected host.

### Conflict of interest statement

The authors declare that the research was conducted in the absence of any commercial or financial relationships that could be construed as a potential conflict of interest.
